# Ambidexterity

**Published:** 1897-07

**Authors:** Herbert Lake

**Affiliations:** Mitchell, Ont.


ARTICLE II.

### AMBIDEXTERITY.

BY HERBERT LAKE, L. D. S., MITCHELL, ONT.

Ambidexterity: a term applied when the movements and sensations of the limbs on one side of the body are under equal control and command with those on the opposite side. There seems to be an unwritten and yet unaltered law to the effect that man should use one hand rather than the other as the instrument of his will. That is to say, one of his hands is the active or controlling member in all that he does.

Right-handedness means, the right hand is the guiding hand, while in work the left one assists. The union is inseparable. In all work which requires both hands the left does as much work as the right, but only as an assistant, and that involuntarily. The active hand is always nearest the work. The baseball player who bats righthanded holds the bat over his right shoulder, has his right hand next the end with which he hits the ball.

The foregoing may also be said of a left-handed person. There are ideas prevalent as to the cause of the majority of about fifty right-handed persons to one left-handed. Some writers affirm that it is a matter of training; that to the untrained child there is no distinction, and as the brain develops, the powers of imitation, together with the training of nurse and mother, bring about the result. There is much in this story Take a child before the age of training can begin; hold before it a simple toy. Which hand will be reached out to grasp it ? As often the one as the other. Further, say for argument's sake that the child is developing left-handedness. Imprison that hand, and see if the child will not adopt the usage of the other. Naturally it will, all theories to the contrary. Set free the imprisoned hand, and what is the result? In one case which came under the writer's notice, the happy faculty of being ambidextrous.

There are many degrees and shades of right and left handedness prevalent in adult life. There are many who are ambidextrous naturally—many to certain degrees showing the natural preference for either the one or the other hand, and yet are indifferent even to preferring the alternate use of both hands. They throw a ball right-handed, bat it left-handed, spring into a race from the left foot, but in the running jump may leap from the right foot. The lower limbs are less closely controlled by the will than the upper. Their movements are more often involuntary, though the moment they are in such a position as to change them from their natural bent the brain is at once called in for a decision. Some writers have attempted to prove that the right hand is the one intended by the economy to be the useful hand, and have gone so far as to say that left-handedness is indicative of a lower degree of intelligence. The idea is combatted by all the observation and tests one can bring to bear on the subject.

We know that where there is a choice of two directions of growth or movement in plants or animals without apparent advantage either way, a preference is shown for the one over the other. For example, among the heavenly bodies the planets and their accompanying satellites follow a similar law; all accompany the earth and its moon save the moons of Uranus, which circle their primate an opposite way. The woodbine twines in its own direction, possibly from left to right, but the climbing hop-vines may reverse that and run from right to left. The grain of the rock-elm is always from left to right, but the ironwood of the northern zones grains in the opposite direction. The squirrel will turn to the right as he spirals up a tree, unless there is danger in that direction; but his cousin the chipmunk almost invariably seeks the opposite course. When the question of moral intelligence is mooted in this connection, what can be said of the coon, who turns neither to the right nor left as he shins up out of danger, his heart full of guile and his stomach full of stolen property ?

Why does man use his right hand more than his left ? Can the arrangement of the internal organs be made to account for it ? The heart is lying obliquely in the left side. The organs of respiration differ in size and weight. The organs of respiration differ in size and weight. The blood vessels in the upper part of the body are unsymmetrically arranged. More than one scientist has undertaken to prove this a cause for the action, though it is hard to see that, separate from the brain, any of the organs have aught to do with it, Now; the left side of the brain controls the limbs on the right side, and *vice versa*. Does any one venture to say that the left brain is greater in size, weighs heavier, or contains more matter than the right ? Popular usage, early training and a predisposition to the right hand accounts for the majority of right-handed persons. It is a fact, however, which cannot be gainsayed, observation has established, and tests have proven that a left-handed person is with that left hand more skilful, more steady than is a right-handed one. It is also a fact that a left-handed person is ambidextrous to a greater extent than is a right-handed one. Popular prejudice is against the left hand, and the right is trained to dexterity. Manufacturers of instruments, of utensils, etc., do not allow for left-handedness, and as a result the unfortunates have either to work under a handicap or adapt themselves to the circumstances.

What is the benefit to a dental operator in being ambidextrous? What operations has he to perform in which he would be helped by his ability to use either hand with equal skill? The statement is ventured, and though discussable, is nevertheless made, that instead of being of use ambidexterity would be a hindrance. Time is money to the dentist, and the time lost in selecting instruments, shifting positions, etc., would not suffice to recompense for the advantage gained by the use of the opposite hand. Again, the servant may make a good master, but the master never makes a good servant. The right hand of the operator is the directing, the motive hand. The left is the assistant. A gold filling is in progress. While the right hand is placing the pellets in position and guiding the mallet, the left is adjusting the rubber-dam, managing the saliva pump, holding the mouth mirror, etc.,—a deal busier than the right hand, but still occupying a minor position. Now, let us change places. The left hand directs the work, and training and practice will insure a degree of success unsuspected, but no amount of will-power, practice or determination will enable the operator to perform with the right hand the work of assistance so admirably and yet involuntarily undertaken by the left. You may make the left hand a successful director, but never will the right occup a subservient position.

As one who has practiced ambidexterity with the desire to benefit thereby, let me advise leaving it alone. The results are no reward for the time spent.—*Dominion Dental Journal.*

